# Supplementary figures and images for: CFTR Lifecycle Map—A Systems Medicine Model of CFTR Maturation to Predict Possible Active Compound Combinations
Source: Int J Mol Sci. 2021 Jul 15;22(14):7590. doi: 10.3390/ijms22147590 (PMC8306775; doi:10.3390/ijms22147590)

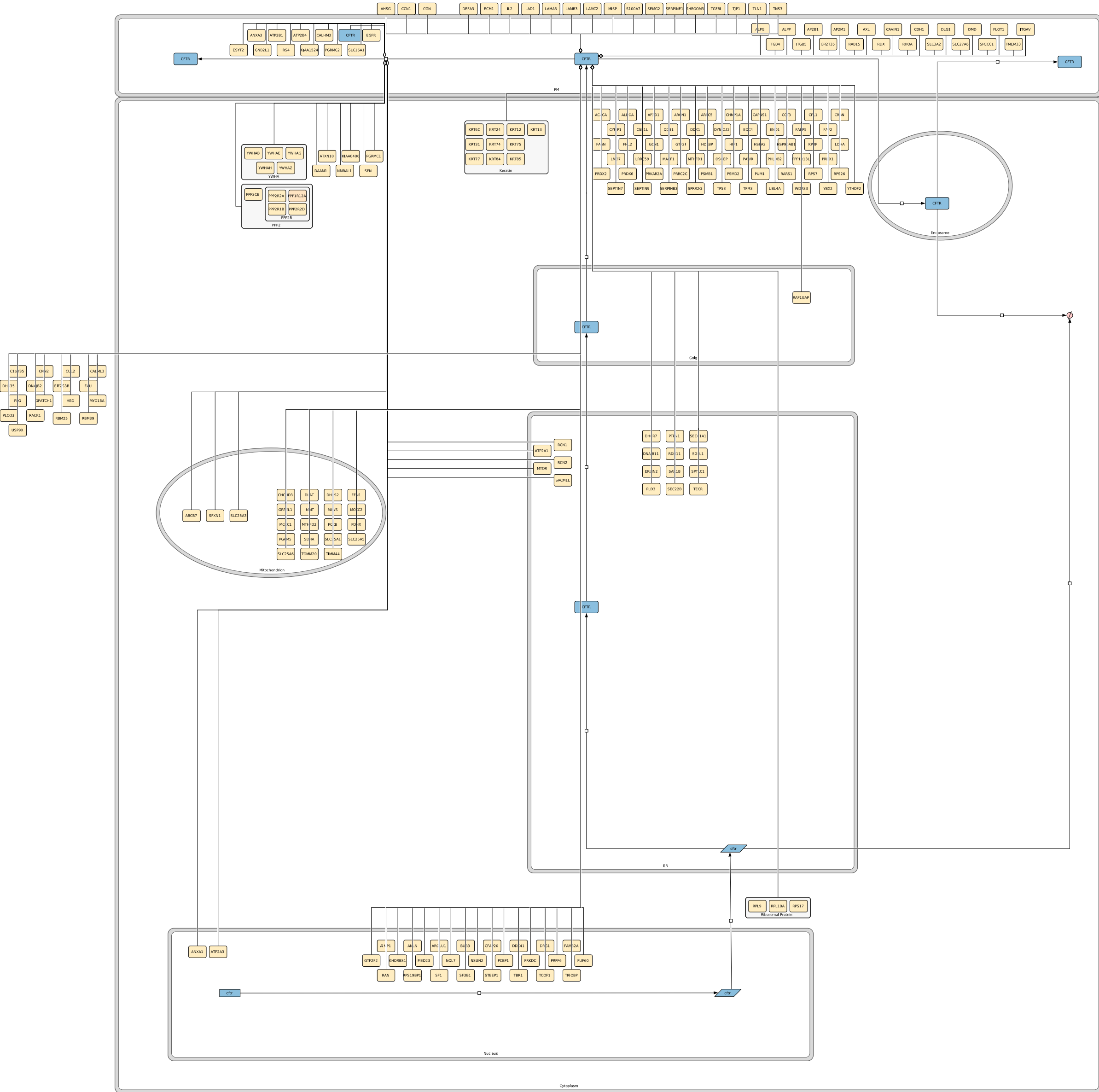

Supplement: Supplementary file 1 [file ijms-22-07590-s001.zip › CoarseMaps/Activity_HT.pdf]

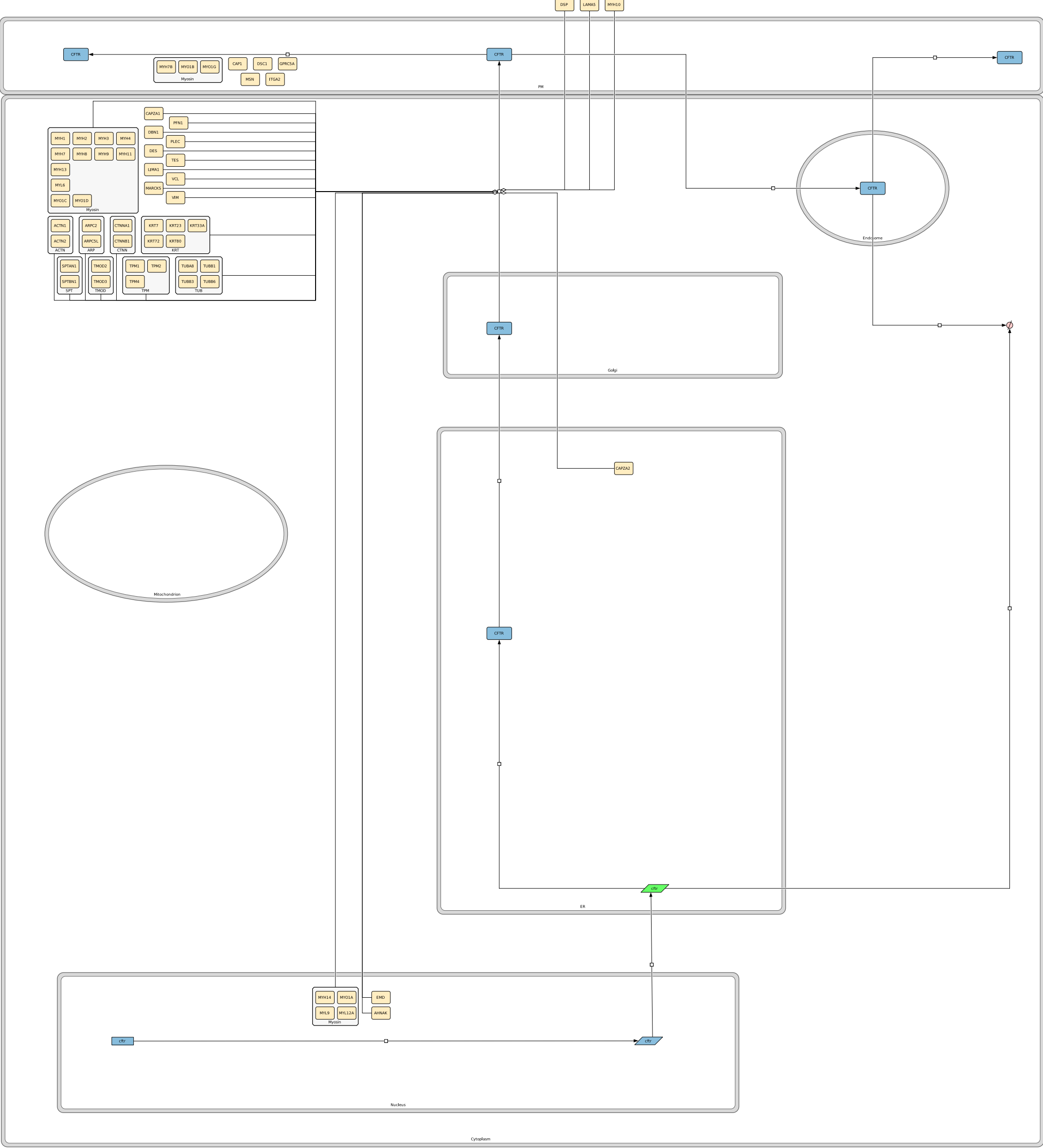

Supplement: Supplementary file 1 [file ijms-22-07590-s001.zip › CoarseMaps/Cytoskeleton_HT.pdf]

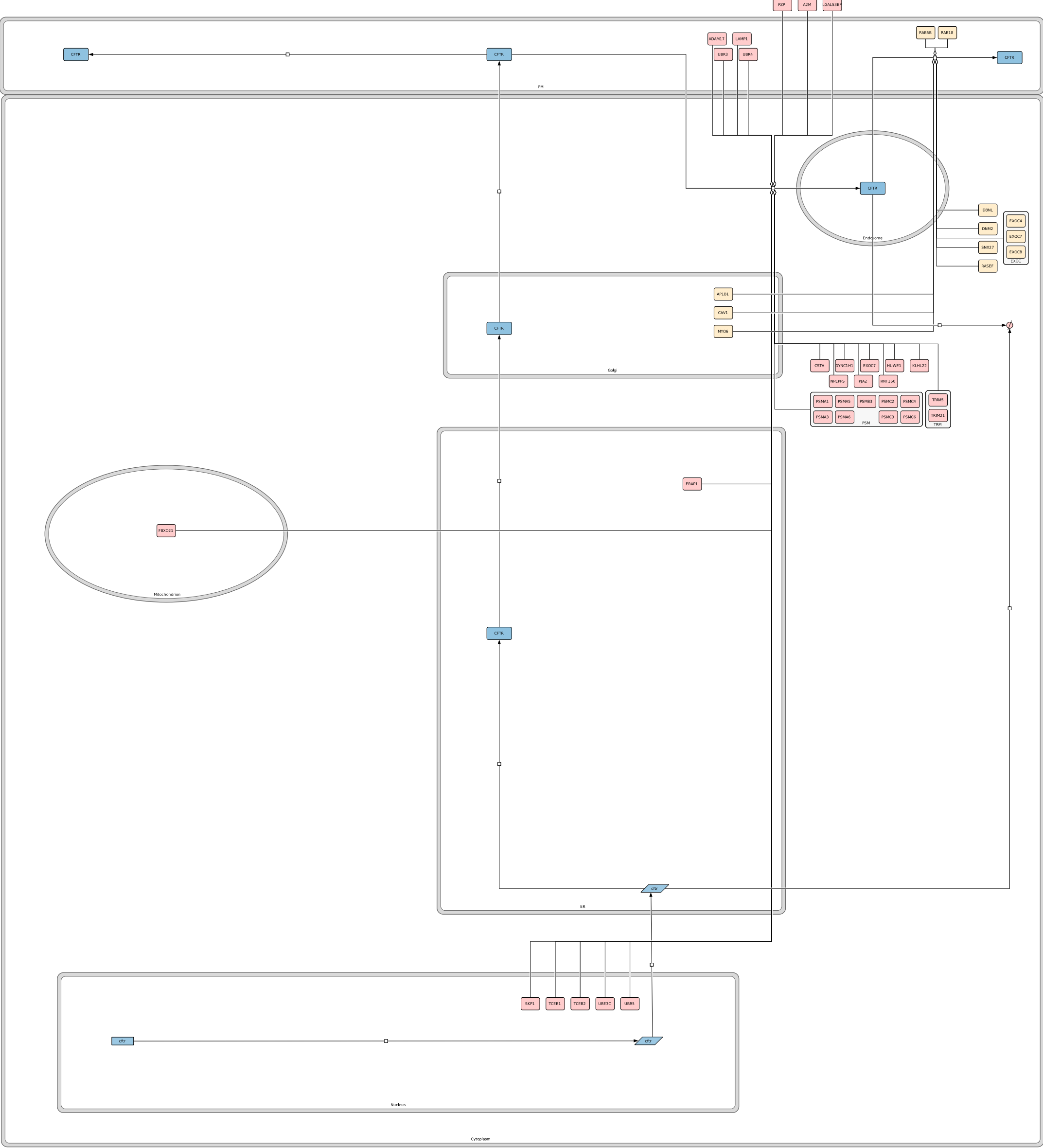

Supplement: Supplementary file 1 [file ijms-22-07590-s001.zip › CoarseMaps/Endocytosis_HT.pdf]

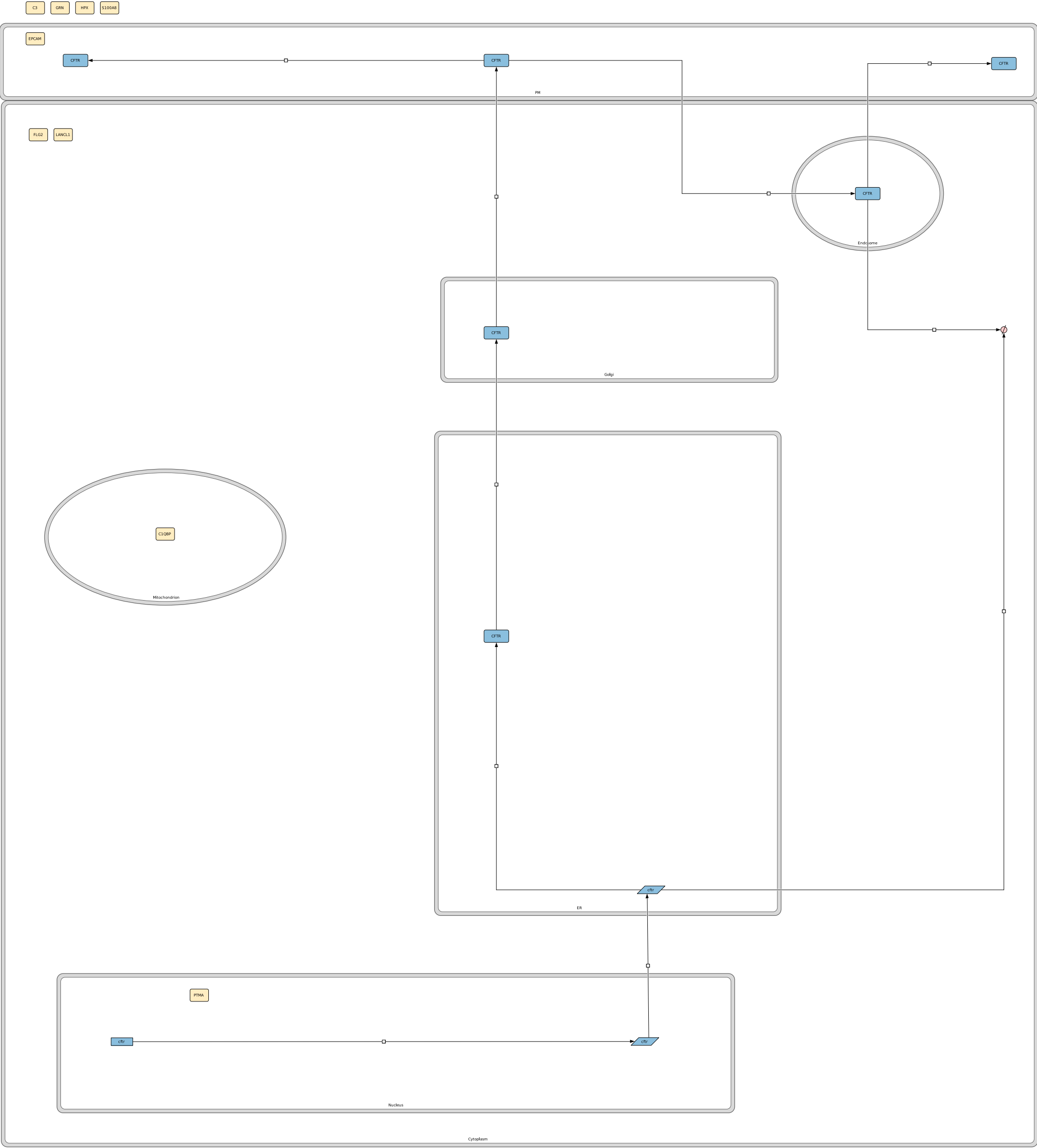

Supplement: Supplementary file 1 [file ijms-22-07590-s001.zip › CoarseMaps/Immune_HT.pdf]

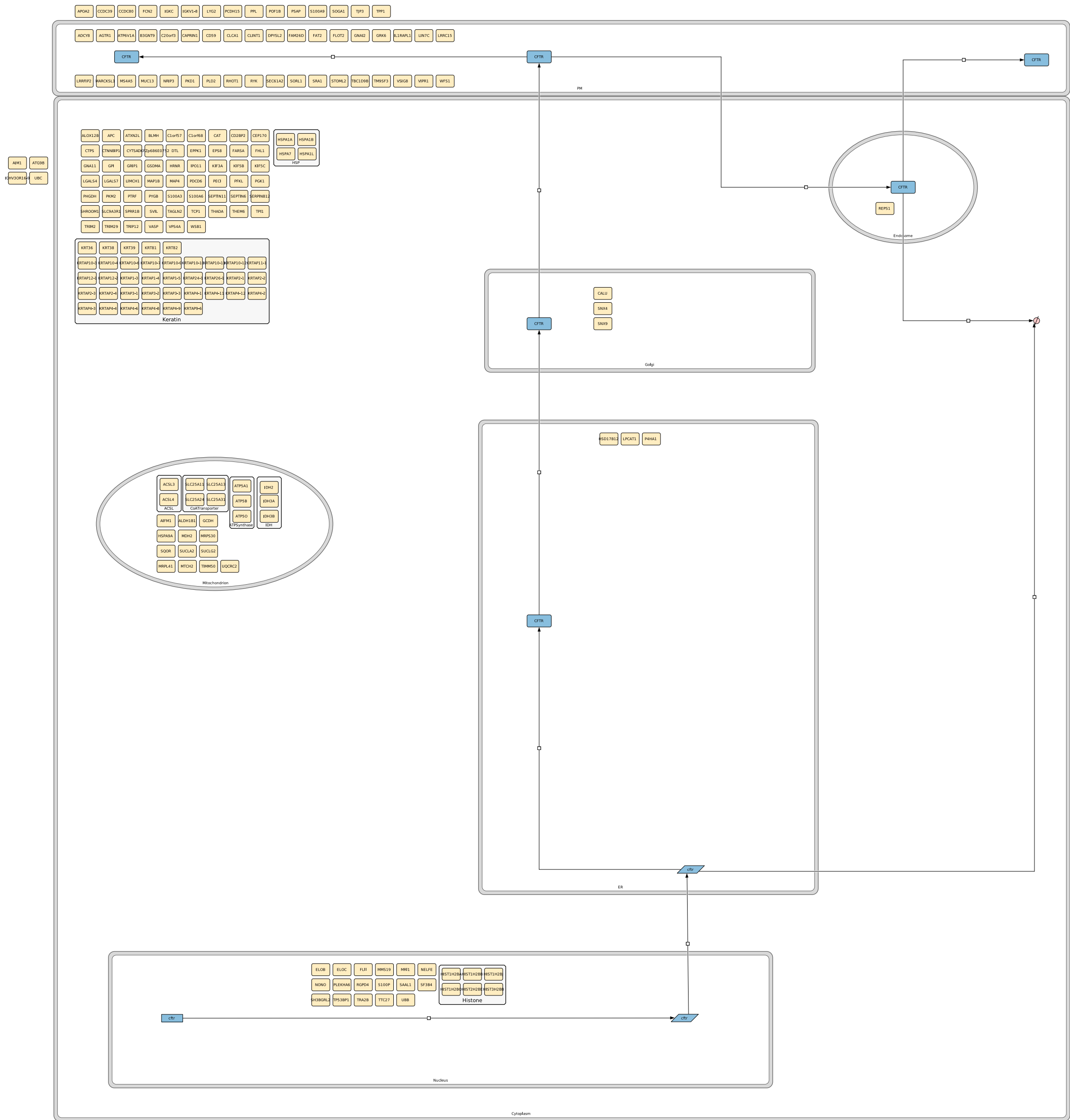

Supplement: Supplementary file 1 [file ijms-22-07590-s001.zip › CoarseMaps/Other_Unknown_HT.pdf]

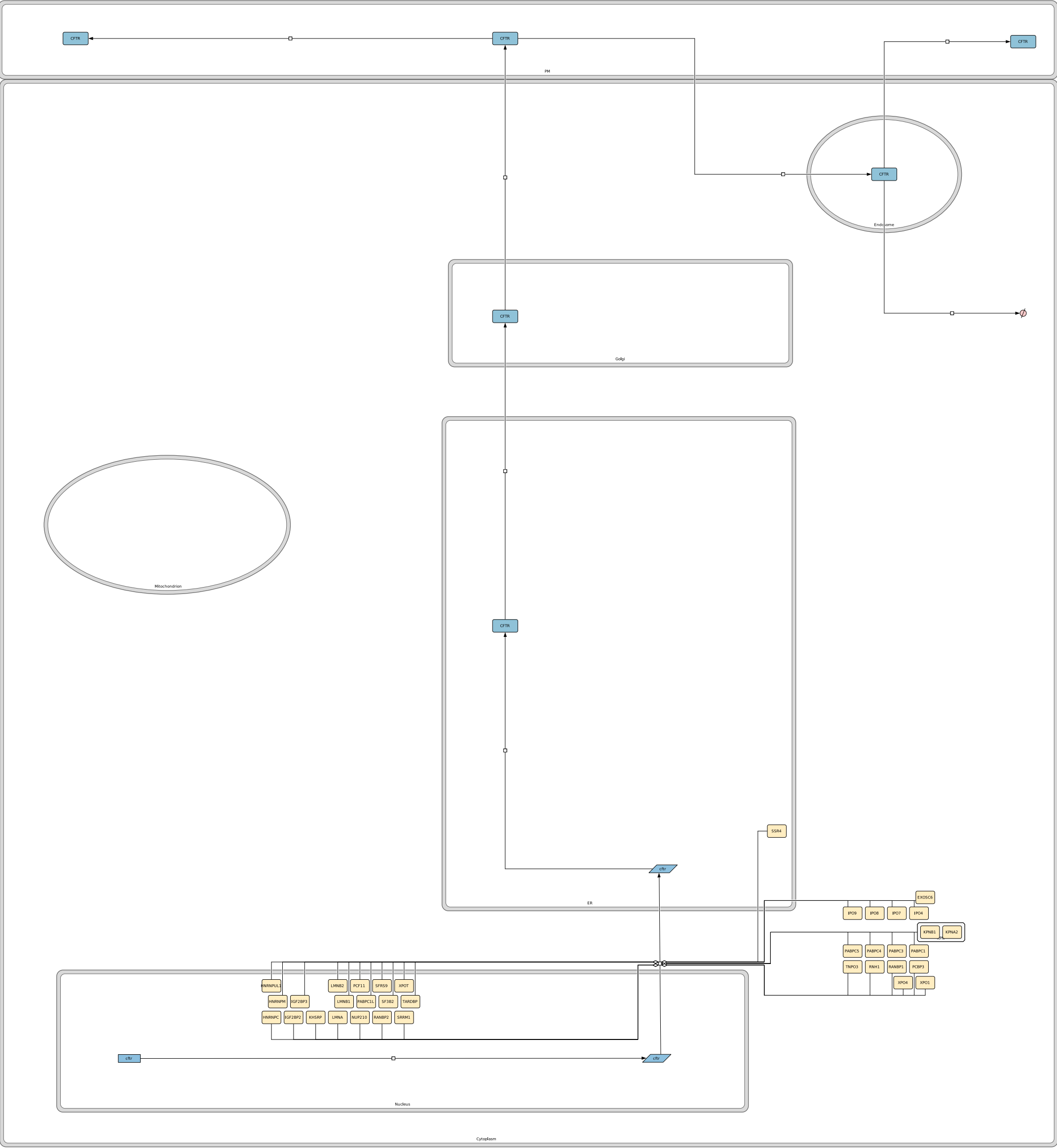

Supplement: Supplementary file 1 [file ijms-22-07590-s001.zip › CoarseMaps/RNAProcessing_HT.pdf]

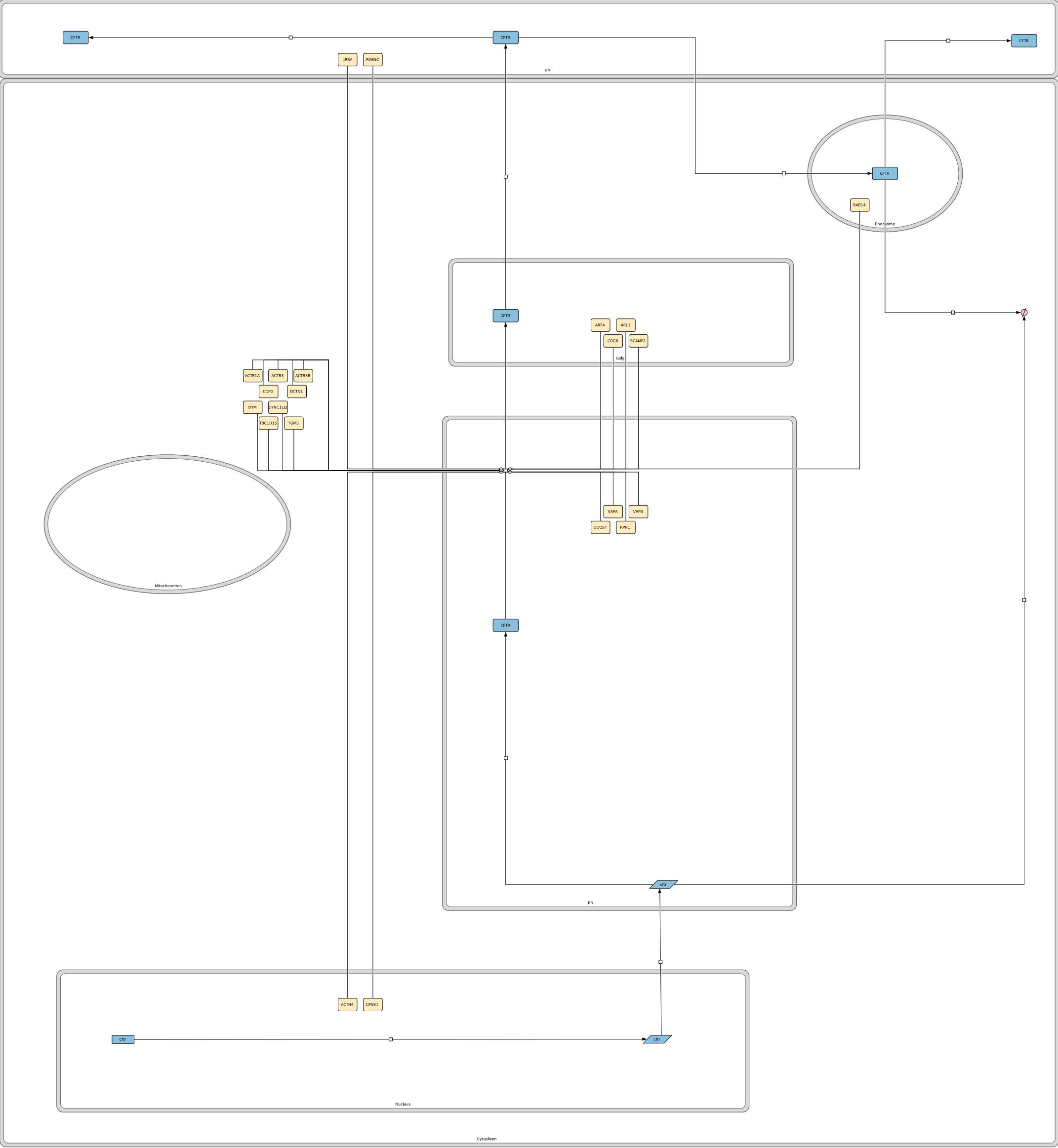

Supplement: Supplementary file 1 [file ijms-22-07590-s001.zip › CoarseMaps/Trafficking_HT.pdf]

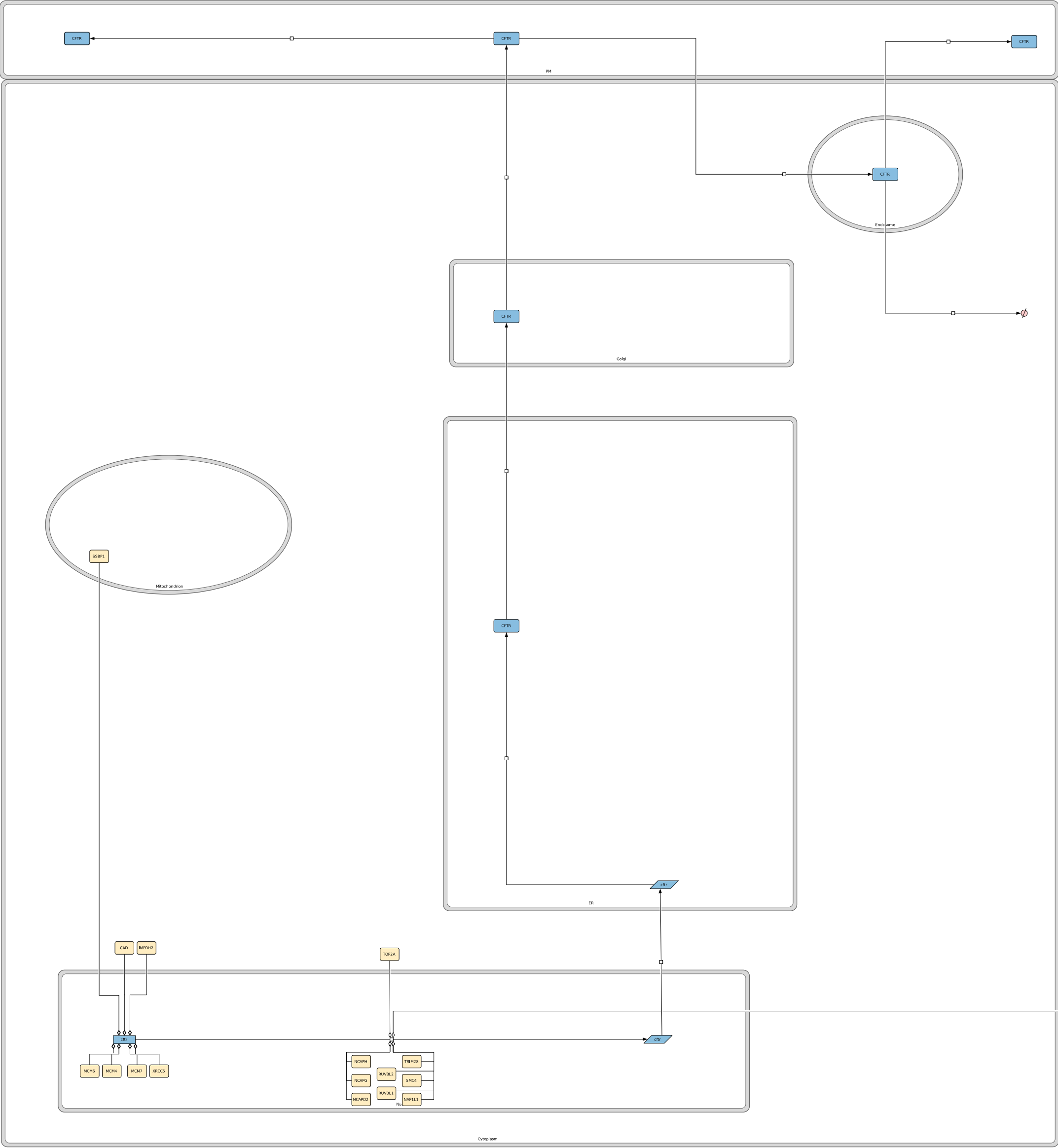

Supplement: Supplementary file 1 [file ijms-22-07590-s001.zip › CoarseMaps/Transcription_HT.pdf]

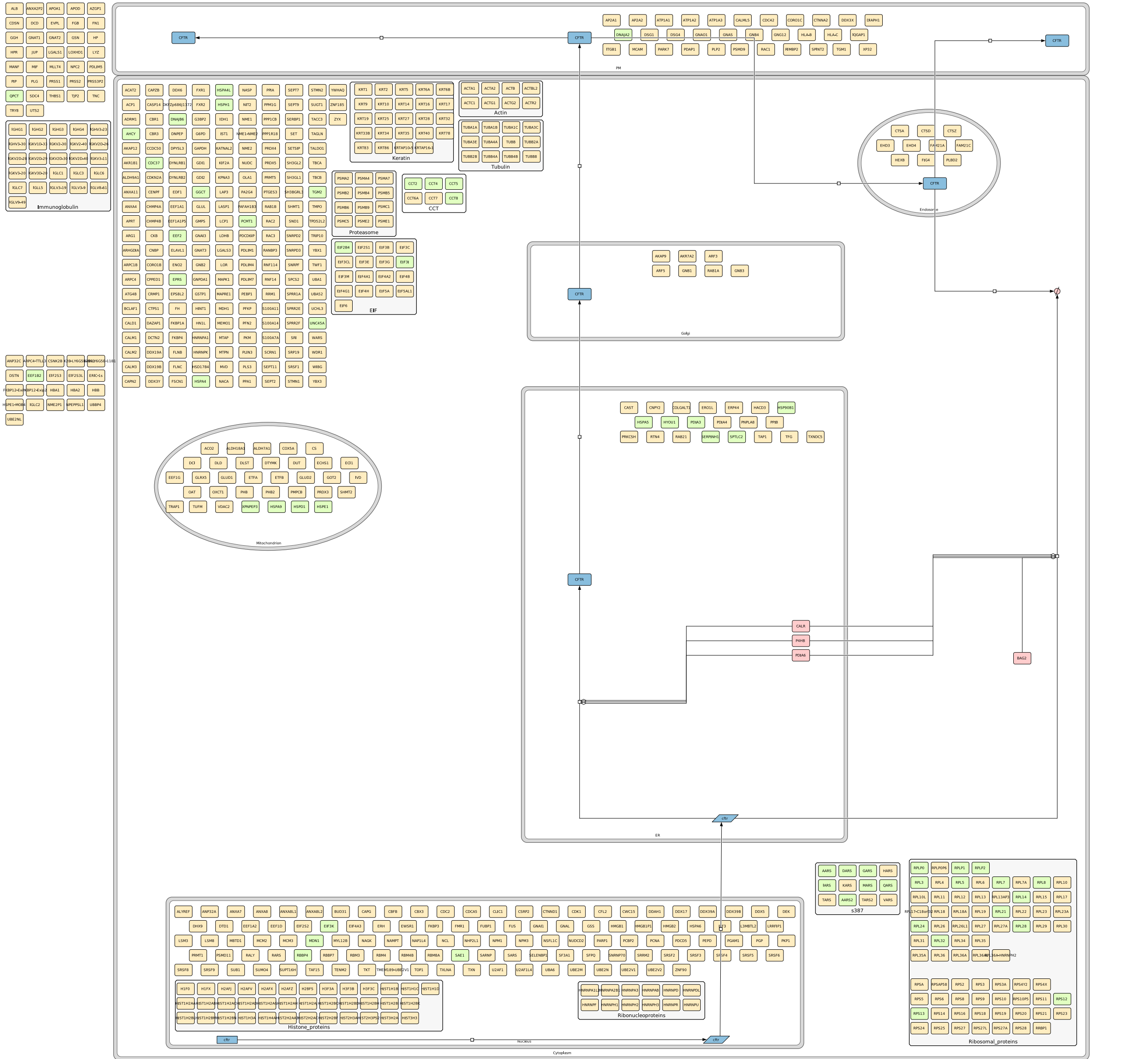

Supplement: Supplementary file 1 [file ijms-22-07590-s001.zip › CoarseMaps/Translation_Folding_QC_HT.pdf]

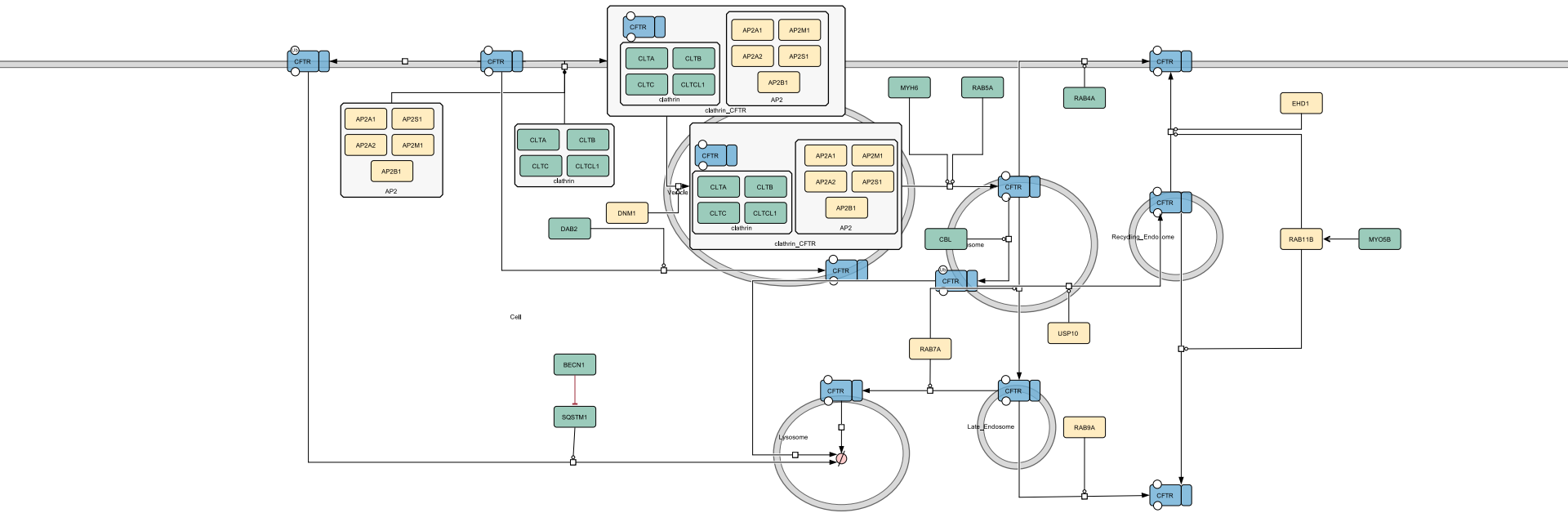

Supplement: Supplementary file 1 [file ijms-22-07590-s001.zip › CoreMaps/Endocytosis.pdf]

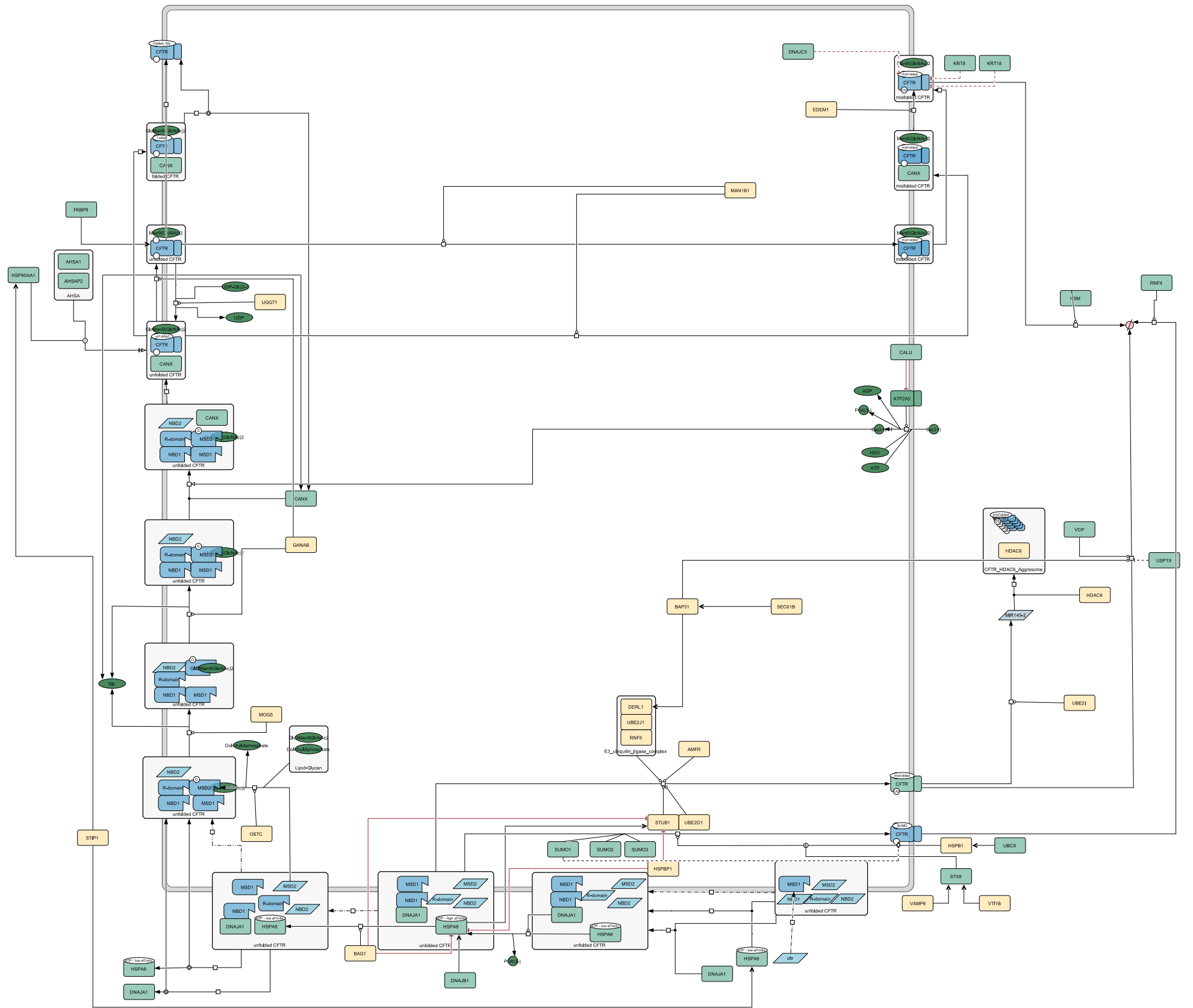

Supplement: Supplementary file 1 [file ijms-22-07590-s001.zip › CoreMaps/ER.pdf]

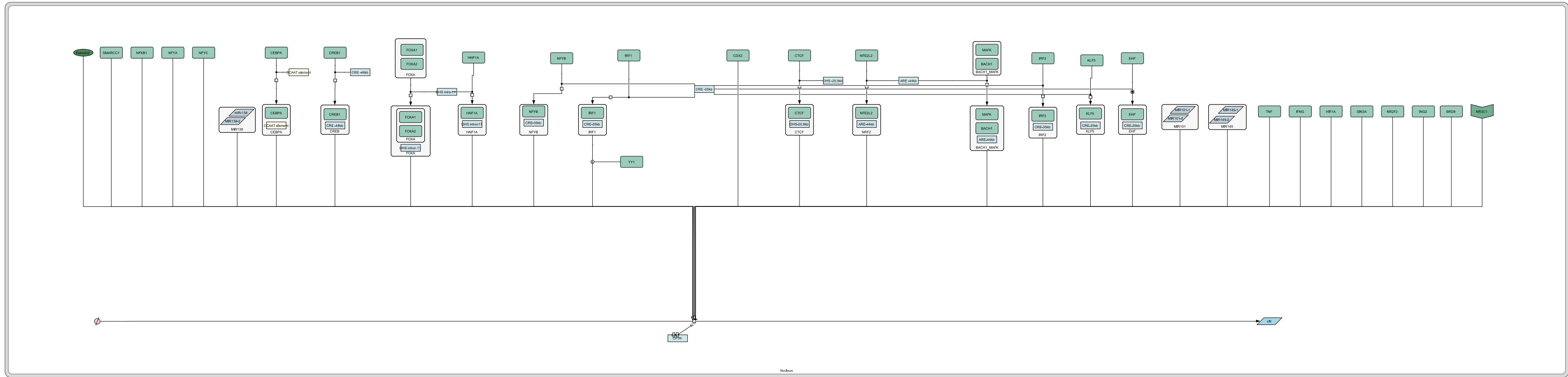

Supplement: Supplementary file 1 [file ijms-22-07590-s001.zip › CoreMaps/Nucleus.pdf]

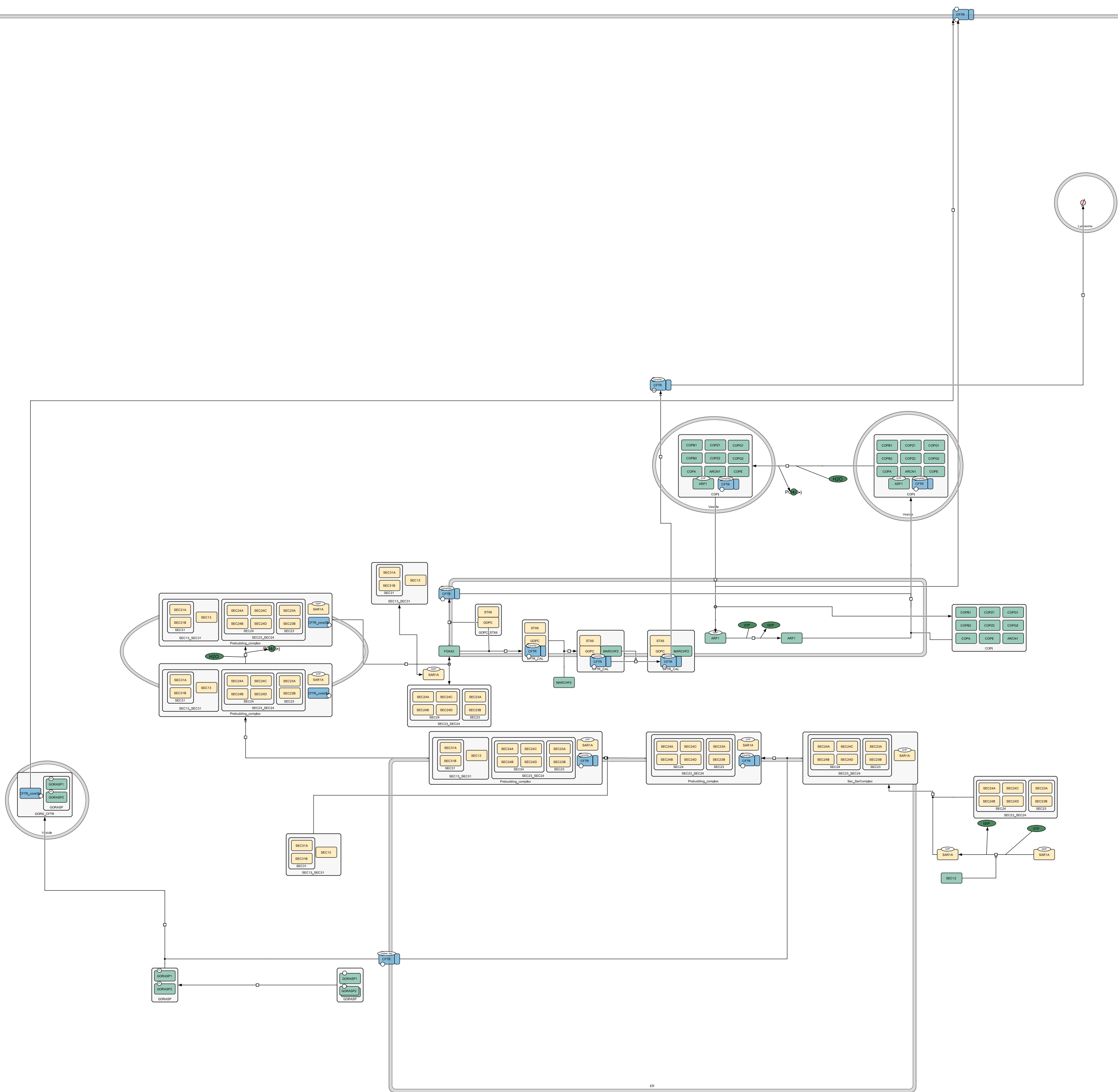

Supplement: Supplementary file 1 [file ijms-22-07590-s001.zip › CoreMaps/Trafficking.pdf]

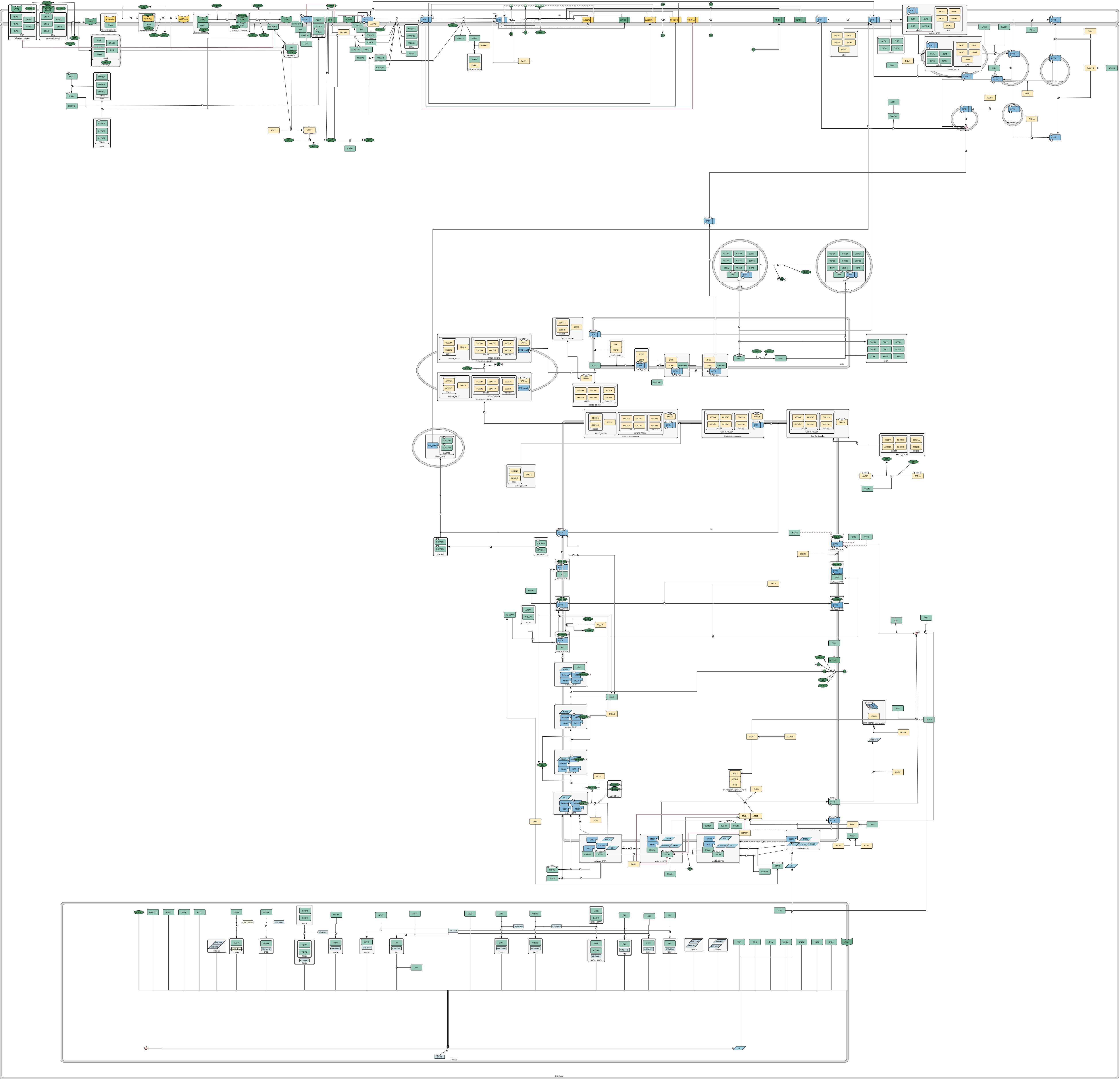

Supplement: Supplementary file 1 [file ijms-22-07590-s001.zip › CoreMaps/WholeCell.pdf]
