# Supplementary material for: CFTR Lifecycle Map—A Systems Medicine Model of CFTR Maturation to Predict Possible Active Compound Combinations
Source: Int J Mol Sci. 2021 Jul 15;22(14):7590. doi: 10.3390/ijms22147590 (PMC8306775; doi:10.3390/ijms22147590)
Supplement: Supplementary file 1 [file Activity_Regulation.pdf › CoreMaps/Activity_Regulation.pdf]

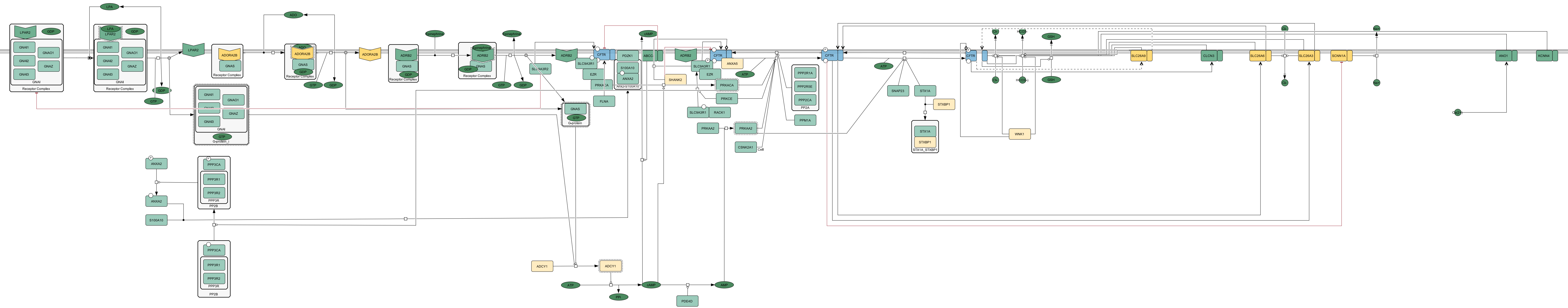

LPAR2
GDP
GNAI1
GNAI2
GNAI3
GNAO1
GNAZ
GNAI
Receptor Complex
LPA
LPAR2
GDP
GNAI1
GNAI2
GNAI3
GNAO1
GNAZ
GNAI
Receptor Complex
GDP
GTP
LPAR2
GNAI1
GNAI3
GNAO1
GNAZ
GNAI
GTP
G-protein_i
ADORA2B
GNAS
Receptor Complex
ADO
ADO
ADORA2B
GNAS
GDP
Receptor Complex
GTP
GDP
ADORA2B
ADRB2
GNAS
GDP
Receptor Complex
Epinephrine
Epinephrine
ADRB2
GNAS
GDP
Receptor Complex
Epinephrine
GTP
GDP
ADRB2
GNAS
GTP
G-protein
CFTR
SLC9A3R1
EZR
FLNA
PDZK1
S100A10
ANXA2
cAMP
SHANK2
ADRB2
CFTR
ANXA5
SLC9A3R1
EZR
ATP
PRKACA
PRKCE
SLC9A3R1
RACK1
PRKAA2
PRKAA2
CSNK2A1
Cell
PPP2R1A
PPP2R5E
PPP2CA
PP2A
PPM1A
CFTR
ATP
SNAP23
STX1A
STXBP1
STX1A
STXBP1
STX1A_STXBP1
CFTR
Cl-
HCO3-
GSH
Cl-
HCO3(-)
GSH
WNK1
SLC26A9
CLCN3
SLC26A6
Cl-
Cl-
SLC26A3
SCNN1A
Na+
Na+
ANO1
KCNN4
Ca(2+)
ANXA2
ANXA2
S100A10
PPP3CA
PPP3R1
PPP3R2
PPP3R
PP2B
PPP3CA
PPP3R1
PPP3R2
PPP3R
PP2B
ADCY1
ADCY1
ATP
PPi
cAMP
AMP
PDE4D
